# Neuroscientists as Cartographers: Mapping the Crossroads of Gonadal Hormones, Memory and Age Using Animal Models

**DOI:** 10.3390/molecules15096050

**Published:** 2010-08-31

**Authors:** Heather A. Bimonte-Nelson, Jazmin I. Acosta, Joshua S. Talboom

**Affiliations:** 1 Department of Psychology, Arizona State University, Tempe, AZ 85287, USA; 2 Arizona Alzheimer’s Consortium, Phoenix, AZ 85006, USA; E-Mails: Jazmin.Acosta@asu.edu (J.I.A.); jtalboom@asu.edu (J.S.T.)

**Keywords:** estrogen, progesterone, androgens, cognition, aging

## Abstract

Cognitive function is multidimensional and complex, and research in multiple species indicates it is considerably impacted by age and gonadal hormone milieu. One domain of cognitive function particularly susceptible to age-related decrements is spatial memory. Gonadal hormones can alter spatial memory, and they are potent modulators of brain microstructure and function in many of the same brain areas affected by aging. In this paper, we review decades of animal and human literature to support a tertiary model representing interactions between gonadal hormones, spatial cognition and age given that: 1) gonadal hormones change with age, 2) age impacts spatial learning and memory, and 3) gonadal hormones impact spatial learning and memory. While much has been discovered regarding these individual tenets, the compass for future aging research points toward clarifying the interactions that exist between these three points, and understanding mediating variables. Indeed, identifying and aligning the various components of the complex interactions between these tenets, including evaluations using basic science, systems, and clinical perspectives, is the optimal approach to attempt to converge the many findings that may currently appear contradictory. In fact, as discoveries are being made it is becoming clear that the findings across studies that appear contradictory are not contradictory at all. Rather, there are mediating variables that are influencing outcome and affecting the extent, and even the direction, of the effects that gonadal hormones have on cognition during aging. These mediating variables are just starting to be understood. By aligning basic scientific discoveries with clinical interpretations, we can maximize the opportunities for discoveries and subsequent interventions to allow individuals to “optimize their aging” and find their own map to cognitive health as aging ensues.

Table of Contents:

1. Why the study of cognitive aging is important: An introduction
2. Gonadal hormones, spatial cognition, and aging: A tertiary model
3. Aging and spatial cognition: A gonadal hormone perspective
3.1. Menopause
3.2. Age-related changes in spatial cognition
3.3. Specific “internal secretions” as mediators of various cognitive functions
3.4. Rodents as a clinically relevant model to test hormone loss and treatment
3.5. Impact of estrogens and progesterone on cognition
3.5.1. Ovarian hormone loss and cognition
  3.5.2. Estrogens and cognition
3.5.3. Progestins and cognition
3.6. Impact of androgens on cognition
3.7. Impact of gonadotropins on cognition
4. Gonadal hormones, spatial cognition and aging: Postulated brain mechanisms
4.1. Synaptic plasticity in cognitive brain regions
4.2. Basal forebrain cholinergic neurons
4.3. Genomic and non-genomic actions
4. Neuroprotective and neurotrophic effects
5. Scopes of research within the tertiary model: Prior journeys, future directions
6. A final comment: How understanding the crossroads of gonadal hormones, memory, and age can help draw a map to optimal brain aging

## 1. Why the Study of Cognitive Aging Is Important: An Introduction 

In the United States, the proportion of the population that is over 65 is increasing. Today, about 12% of the population is over 65, and this percentage is expected to substantially increase to 20% by the year 2020 due to aging of the “baby boomer” generation born in the years immediately after WW-II [1]. As individuals age, it is well documented that some memory loss is observed [2,3]. Learning and novel memory formation allows adaptability in an organism. It allows acquisition and updating of knowledge and skills within different and overlapping neurobiological domains [4]: strangers become familiar friends, new facts are learned, and skill-sets for playing sports, musical instruments, cooking or computer programs are acquired. Within the specific domain of spatial navigation, an individual learns to navigate through a novel environment so that a route to the target eventually becomes familiar, and cues in the environment form associations to help with overall navigation. For a human, this allows one to learn to find their way to a new coffee shop, and, after learning occurs, to navigate to this new location whether one starts the journey from home or the grocery store. For an animal, this allows it to learn the route to a new food source or reward, regardless of its starting location. 

While progression into old age is sometimes associated with a decreased proficiency in learning, learning still occurs within many types of cognitive domains. Indeed, decades of research have resulted in the general consensus that not all domains of learning and memory are equally affected by aging. Normally aged people usually remember how to drive an automobile and brush their teeth (non-declarative memories) and the name of the President (semantic memories), but demonstrate declines in recall of personal experiences and events (episodic memory); episodic memory typically shows greater age-associated decline than semantic memory [2,5]. It is generally thought that by the fifth decade of life in humans, learning occurs more slowly for more difficult cognitive functions, such as that tested on the declarative memory delayed recall task [6,7]. Perhaps most considerably altered with age is spatial learning and memory. Spatial cognition depends upon medial temporal lobe structures such as the hippocampus, and involves the ability to navigate effectively through an environment including acquiring, integrating and retaining environmental features such as landmarks and other prominent cues [8]. Since the historic work of Tolman, learning to navigate through a new environment has been referred to as the formation of a cognitive map [9]. The hippocampus facilitates the formation of a cognitive map and declarative memories, as well as the knowledge of facts and their relationships [10,11], and the neocortex is the location of long-term memory storage [11]. Additionally, working memory, requiring manipulation of information kept “on-line”, is intimately related to the frontal cortex, and is also affected by normal aging revealing a memory decline that becomes more severe as task difficulty increases [5]. Notably, both the hippocampus and the frontal cortex appear to be particularly vulnerable to age-related changes [12,13].

Spatial learning and memory requires an individual to navigate through space to locate a goal. This can be readily tested in rodent models. Many experimental tests of rodent spatial memory aim to assess *working memory*, a form of short-term memory requiring a subject to retain spatial information which must be updated and is useful for only a short period of time [trial-specific information, 14]. In general, working memory is distinguished from *reference memory*, which is necessary to remember information that remains constant over time [task-specific information, for discussion, see 15]. Rodent studies agree with human studies that spatial navigation ability decreases in normally-aged individuals, for both spatial working and reference memory, and that there is individual variability in age-related cognitive change [4]. Many researchers studying aging effects on learning and memory divide aged rodent subjects into impaired and non-impaired groups to compare differences in the groups for various physiological measurements [16,17,18,19,20,21,22,23,24,25]. While the operational definitions for these classifications are not homogeneous among studies, as some classify aged-impaired subjects as those with scores that are beyond the mean of the young group by one standard deviation [17,26], two standard deviations [16,19,22,23], or outside the range of the young [24,25], they all concur that individual expression of age-related memory change exhibits a large range of variability. How individual variability in cognitive prowess occurs during aging is a topic of intense inquiry and is likely due to many variables. Indeed, age-related decline in learning and memory, including hippocampal-dependent spatial function, is embedded within the context of an individual’s life experiences and exposures. These experiences and exposures span many individual and interactive factors which will not be discussed in their entirety here, including, but not limited to, genetic predisposition, early organizational effects of gonadal hormones, enriching or stressful experiences, and dietary factors (e.g., choline exposure). 

The main tenet driving discussion in the current review is the relation between gonadal hormones, memory and age, with the hypothesis that gonadal hormone milieu impacts cognition during aging. These effects appear to depend upon a myriad of complexities regarding the *when’s, how’s* and *why’s* of gonadal hormones’ impact on cognition. Scientists are just beginning to understand the incredible diversity of effects that gonadal hormones can have on the central nervous system, as well as the great impact these exciting discoveries could have on our understanding of brain health as aging ensues. 

The goal of this review is to discuss the most recent findings of gonadal hormone effects on cognitive aging, in the context of the landmark gonadal hormone-related discoveries that have provided the framework to establish a path to healthy cognitive aging. Animal models have been widely used in this research area because they allow methodical experimental evaluation while controlling for, or obviating, factors likely influencing outcome in clinical studies such as exact age, duration of hormone loss or replacement, specific type and dose of hormone manipulated, socioeconomic status and education. Rather, in animals studies the majority of these variables, and their interactions, can be systematically tested and used to compliment and aid understanding of the clinical studies. Because spatial cognition has been the focus of the majority of the basic science research in this area, spatial cognition will, here to, be the primary focus.

## 2. Gonadal Hormones, Spatial Cognition, and Aging: A Tertiary Model

**Figure 1:**
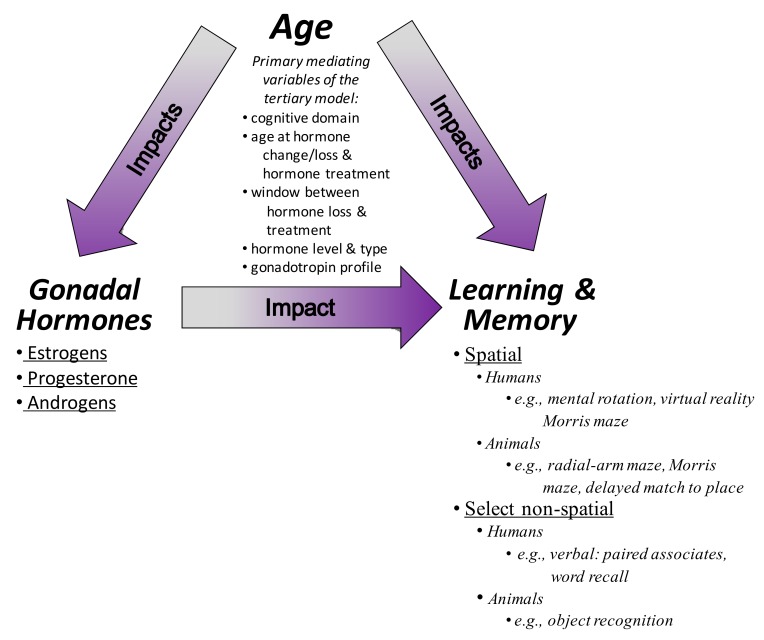
Tertiary model representing interactions between gonadal hormones, memory and age.

Several decades of research have converged to indicate that gonadal hormones are potent modulators of brain structure and function, including in brain regions known to be intimately linked to learning and memory. Many of these cognitive brain regions are sensitive to changes as aging ensues. As represented in Figure 1, there is a tertiary model representing interactions between gonadal hormones, spatial cognition and aging, wherein research within each domain has identified strongly supported tenets: 1) gonadal hormones change with age, 2) age influences spatial learning and memory, 3) gonadal hormones influence spatial learning and memory. 

While it is clear these individual tenets are strongly supported by empirical data, a fundamental question in aging research regarding interactions has yet to be clearly answered. “How do gonadal hormone changes that occur with age relate to memory changes that occur with age?” How the presented three tenets interact with each other, and their mediating variables, is an important area of research that will yield insight into life exposures that influence age-related brain and cognitive change to ultimately impact the cognitive phenotype of the aged individual. Below we discuss each of these three tenets in turn, with examples of interactions and mediators presented when available from the literature.

## 3. Aging and Spatial Cognition: A Gonadal Hormone Perspective

### 3.1. Menopause

Inherent to any discussion of gonadal hormones and aging in the female is menopause. Menopause, occurring typically in the fifth decade of life, is characterized by loss of ovarian-derived circulating hormones, including estrogen and progesterone [27].

Research transitioning to each point has been strongly supported by empirical data: 1) aging impacts gonadal hormone milieu, 2) aging impacts learning and memory, 3) gonadal hormones impact spatial learning and memory. How these three tenets interact is a key area of research that will yield insight into life experiences and exposures that influence age-related brain and cognitive change to ultimately impact phenotype of the aged individual. Also noted are several likely mediating variables, as discussed in this review and supported by the literature, as well as some examples of cognitive measures evaluated in research studies.

The majority of women undergo menopause not from oophorectomy (*i.e.*, removal of the ovaries), but as a transitional hormone loss following age-related alterations of the hypothalamus, pituitary and ovary, ultimately resulting in ovarian follicular depletion [27]. Early in the aging process, neuronal changes in the hypothalamus are hypothesized to initiate transition into reproductive decline, leading to reproductive senescence [28]. The ultimate hormone profile of the older reproductively senescent female rat and woman differ, limiting the use of the normally aging ovary-intact female rat as an optimal model of human menopause. Nonetheless, the rodent model provides exciting and illuminating insights to understand mechanisms of menopause itself since there are some commonalities regarding reproductive physiology [28]. As aging ensues in women, estrogen and progesterone decline due to decreased ovarian follicular reserves [27]. Thus, ovarian follicle depletion ultimately causes hormone loss during menopause in women. In contrast, the aging rat undergoes estropause, a persistent estrus state due to chronic anovulation rendering intermediate estrogen levels, or a pseudopregnant/persistent diestrus state characterized by high progesterone levels due to increased ovulation and corpora lutea [29]. These changes in ovarian-derived hormone release in the rat are primarily due to hypothalamic/pituitary axis alterations [29]. Thus, the primary mechanism that ultimately results in reproductive senescence and circulating hormone alterations in the woman is ovarian follicle depletion, while in the rat it is the hypothalamic-pituitary axis. 

### 3.2. Age-related changes in spatial cognition

Across multiple species, including humans [30,31], monkeys [32], dogs [33] and rodents [34,35,36], performance on spatial navigation tasks declines with age, including remembering of spatial cues such as landmarks and other relevant environmental information [8]. In fact, one of the most consistent findings in the animal cognition literature is that aged rodents exhibit poor scores on working and reference memory tasks that require spatial navigation. The majority of the studies testing age-related changes in spatial cognition used male rodents, with studies reporting age-related memory decline on amultitude of tasks using various protocols and procedures [34,37,38,39,40,41,42,43,44,45,46,47,48,49,50,51,52]. In the last decade, there have been increased efforts to study the effects of age-related spatial cognitive decline in female rodents, with the driving force in many studies relating to interest in the relation with concurrent gonadal hormone change. For example, spatial reference memory decline on the Morris maze emerged by 12-16 months of age in ovary-intact rats, when ovarian hormone levels started to change and estropause ensued [36,53].

When evaluating the vast cognitive aging literature in the rodent, age-related changes in spatial learning and memory are multidimensional and complex, with, for example, age-related changes dependent upon which phase of learning is being tested and task demand. Age-related cognitive deficits seem to be greatest during task acquisition, as young and aged rodents eventually reach comparable asymptotic levels on spatial working and reference memory tasks when given extended training [47,54]. This effect may interact with degree of cognitive impairment in the aged animal at the onset of testing sessions [54].

**Figure 2:**
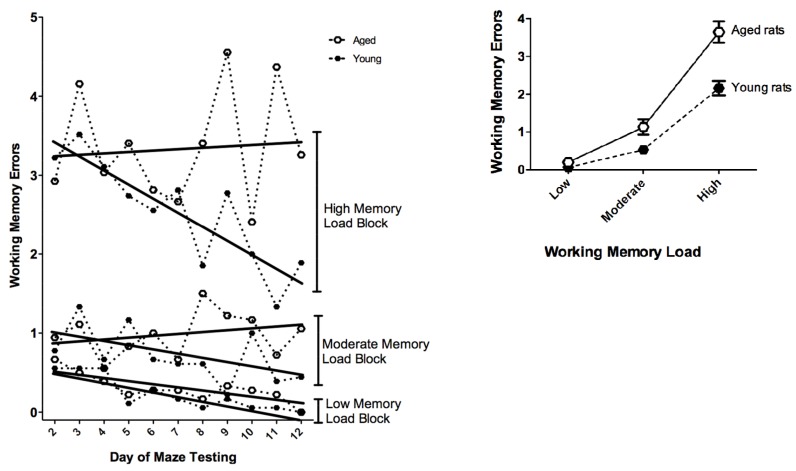
Age alters the ability of animals to handle an increasing working memory load [35]. **a)** Mean ± SE number of spatial working memory errors committed by young and aged rats averaged into Low, Moderate, and High load blocks. Also depicted is the linear trend for each block, for each group. Both Young and Aged groups exhibited significant linear trends on the Low load block (regression equations: Aged: y = -0.042 x + 0.600, Young: y= -0.058 x+.639), while only the Young group showed a significant linear trend on Moderate and High load blocks (ys = -0.058 x + 1.12 and -0.176 x + 3.76, respectively). Findings suggest that both young and aged animals could learn to handle a low memory load and that while the young animals could also learn to handle moderate and high memory loads, the aged animals could not. **b)** Mean ± SE number of spatial working memory errors on Low, Moderate, and High load blocks, averaged across the latter testing days, for young and aged rats. As trials increased, the number of elements of information to be remembered increased. Aged animals committed disproportionately more errors on the latter trials, when the spatial working memory load was highest.

It is noted that age-related deficits have been observed after spatial task acquisition as well. In this context, the greatest age-associated cognitive decrements have been seen when spatial memory demand is high. In humans, age-related deficits are exacerbated with greater working memory complexity, an effect shown on multiple tasks and within non-spatial (e.g., verbal) and spatial domains [55]. In rodents, age-related spatial deficits become more pronounced as memory demand increases. This has been shown for age-associated interference related deficits [43], and for memory capacity deficits [35,56,57,58]. For example, on the spatial water radial-arm maze task, as trials progress within any given session, animals need to hold a greater number of items of information in spatial working memory. Young and aged female rats did not differ in the ability to handle an increasing spatial working memory load during the initial portion of testing (Figure 2) [35]. However, as testing progressed across days young animals learned to handle more spatial working memory information. This resulted in a significant learning curve for young animals on all trials, whether spatial working memory load was low, moderate, or high. In contrast, aged animals exhibited a learning curve only on the earliest trials, when spatial working memory load was low. As spatial working memory load increased, aged rats had difficulty remembering which arms they had visited within a session, and they could not learn to handle this increasing amount of information across testing sessions. Aged rats also made disproportionately more errors on the latter trials, when spatial working memory load was highest. Thus, aged female rats exhibited progressive performance deterioration as the number of items to be remembered, or working memory load, increased (Figure 2).

There is other evidence that aged animals have difficulty sustaining successful performance as other types of memory demands increase, such as when a delay is imposed between trials. Such findings that aged rats exhibit delay-dependent deficiencies in performance is typically interpreted as a faster rate of forgetting [38,59,60,61], although not all studies find age differences in rates of forgetting, which may be related to overtraining or animals reaching criterion performance [38,50,62].

This latter point is underscored by research in humans touching on the boundaries between what is, and what is not, normal cognitive aging. For example, research indicates that if younger and older people learn information to the same level, even though the older individuals may take longer to reach this point, older people will not forget the learned information more rapidly [6,7]. Thus, collectively the literature suggests that while there are clear age-related changes in learning and memory in humans and in animal models, there is great variability across individuals. Moreover, decrements associated with aging are typically seen regarding only certain types of information, with spatial memory one of the most robust deficits noted, as well as an age-related selective vulnerability to acquisition and task components with high demand.

### 3.3. Specific “internal secretions” as mediators of various cognitive functions

By the beginning of the 20th century, specific “internal secretions”, now referred to as steroid hormones, were known to be chemical mediators of the phenotype [63]. Steroid hormones released from the gonads have since been shown to be important for not just classical reproductive actions [64], but also for influencing neural plasticity and brain functions such as learning and memory. In humans and animal models, estrogens, progesterone and androgens have each been shown to impact spatial cognition. To evaluate steroid hormone levels and cognitive effects in humans, researchers have been creative in their assessments and have reported effects: across menopause transition stages [65], with sex-change operations and concomitant sex hormone treatment [66], and before versus after hormone therapy treatment in surgically menopausal women [67]. To experimentally and methodically test the cognitive effects of steroid hormones in rodent or monkey models, the traditional procedure is to remove the source of major endogenous synthesis and release, the testes in the male (gonadectomy, or Gdx) or the ovaries in the female (ovariectomy, or Ovx), and give the exogenous steroid of question as a treatment regimen before testing. 

Over the last decade, both the human and animal literature evaluating the potential influence of gonadal hormones on brain health and function during aging have increased in breadth and depth. Much of this is because of the recent intense discussion and debate about whether hormone therapies impact cognition during normal aging or Alzheimer’s disease (AD). This emanates in part from reports including a meta-analysis that estrogen-containing hormone therapies decrease the risk of AD by 29% [68], that placebo-controlled studies show estrogens improve memory or decrease dementia in female AD patients [69,70,71,72], that menopause exacerbates age-related cognitive changes in several domains, including visuospatial abilities [73], and then more recently, the outcome of the Women’s Health Initiative studies which show null or detrimental effects on cognition or dementia from the most commonly used hormone therapies (for discussion see [74]). Further, the clinical implications and health-related importance of understanding the effects of ovarian hormone loss and replacement has been underscored by realization that the life expectancy of women has increased from an average of 54 years in 1900, to recent values estimating expectancy of about 80 years [75]. Since age of spontaneous menopause has remained stable, women are now living approximately one-third of their lives in a hypo-estrogenic menopausal state [76,77,78]. Currently, it is not clear what ovarian hormone loss, nor ovarian hormone replacement, does to the brain and its function. However, the emerging findings in the literature have been illuminating, diverse, and exciting. They have shown that multiple parameters impact the extent, and even the direction, of cognitive effects of gonadal hormones. This underscores the impact of gonadal hormone effects on brain function and its rich plasticity. The discussion here will not exhaustively cover this immense and growing literature. It will be limited to points necessary to provide context for the discussion of gonadal hormone effects on cognitive aging, with special emphasis on the spatial domain. 

### 3.4. Rodents as a clinically relevant model to test hormone loss and treatment

In the literature evaluating women, one cognitive domain shown to be negatively affected by aging and hormone loss is verbal memory, and there is evidence that some forms of estrogen therapy can ameliorate verbal memory decline [67,74,79]. Verbal memory depends on the integrity of the hippocampus, as demonstrated, for example, by functional neuroimaging [80,81,82], as well as event-related potential recording studies [83,84]. Moreover, positron emission tomography (PET) studies showed that estrogen therapy altered hippocampal regional cerebral blood flow activation patterns, which could be interpreted as enhancing hippocampal function, during verbal memory performance in menopausal women [85,86]. In the female rat, spatial memory is particularly sensitive to aging and ovarian hormone loss, and estrogen treatment can enhance spatial memory [53,57,58,87,88,89,90,91,92,93,94,95,96,97,98,99]. Spatial navigation is hippocampal-dependent, as evidenced by work showing that hippocampal-lesioned animals exhibit deficits in solving spatial memory tasks, effects which are dissociated from other brain regions [100,101,102]. Hence, both verbal and spatial memory, which rely on hippocampal integrity, are compromised with aging in women and in rodents [74,103], and the protective effects of estrogen therapy seem to target the cognitive domains that are most vulnerable to aging and hormone loss in both species. Thus, the connection between aging, brain region, and effects of estrogen therapy suggests that spatial memory in rodents provides a clinically relevant model for cognitive function evaluation of hormone loss and hormone therapy.

### 3.5. Impact of estrogens and progesterone on cognition

#### 3.5.1. Ovarian hormone loss and cognition

Accumulating evidence supports the interpretation that ovarian hormone loss contributes to cognitive decline in women. A recent longitudinal study evaluating 1903 women from 2000-2006 found a decreased cognitive processing speed in women during late perimenopause; these effects were not accounted for by depression, anxiety, sleep issues or vasomotor symptoms [104], while other research has shown clear changes in verbal fluency during postmenopause [105]. Further, in women undergoing the transition to menopause, memory complaints were associated with poorer memory encoding, which was predicted by estrogen levels [106]. Interestingly, the noted transitional menopause-related cognitive change is reported as slight, and seems to vary depending on numerous variables including depression and body mass index [105,106]. Clinical findings also show that women exhibit cognitive decline after surgical menopause [107,108,109], including on global cognitive function as tested 3 or 6 months post-surgery [107]. Further, surgically menopausal women exhibited lower memory scores relative to naturally menopausal women, and age of oopherectomy (surgical ovary removal in the woman) and greater years since surgery correlated with poorer performance [110]. 

Preclinical rodent model evaluations also show that ovarian hormone loss can induce cognitive changes, an effect depending on many factors, including age [53,97,98,99]. Surgical ovarian hormone loss (e.g., Ovx) produces cognitive decrements in young adult female rats on spatial and non-spatial tasks [53,111,112,113,114,115,116]. Before evaluating the effects of surgical menopause in aged animals, we had shown that Ovx impairs water radial-arm maze working memory in young animals [111]. To our surprise, when we tested aged Ovx animals on this task and compared them to Sham animals of the same age, we found Ovx-induced cognitive enhancements [57]. We have since replicated this finding many times, and our research indicates that the Ovx-induced enhancements are likely due to removal of elevated progesterone levels seen with estropause in the rat [57,58,91] (note: these progesterone increases are not seen with menopause in women; see Menopause section 3.1). To date, only four studies have evaluated Ovx effects on maze learning and memory in middle-aged rats [53,97,98,99]. In middle-aged females 12-16 months-old, Ovx did not impact spatial reference memory [53,97,98]or spatial working memory [98,99]. However, spatial working memory deficits were detected in 17 month Ovx old rats following high-demand time delayed memory retention tests [98]. These data suggest that Ovx-related memory changes in middle-age may become evident when working memory demands are more challenging. In this regard, we previously demonstrated that Ovx alters memory in both young and old rats [57,58,91,111], effects which were more pronounced as working memory load increased by escalating the number of items to remember. Thus, elevating working memory demand either by extending time delays to challenge retention, or by increasing the number of items to remember, allows a broader scope of evaluations to realize Ovx-induced memory changes across the ages. It is noted that exacerbated deficits in aged animals are also seen with a higher memory demand, as described above, further indicating that incremental alterations in task demand could yield insights into changes that may not be seen otherwise. 

**Figure 3:**
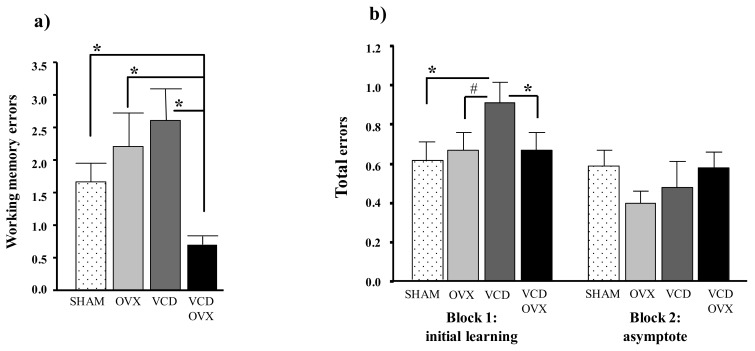
Spatial memory performance in several experimentally-induced forms of ovarian hormone loss, VCD-induced follicular depletion (gradual, transitional menopause) and Ovx (surgical menopause), in the middle-aged rat [87]. Also included was a group that received VCD followed by Ovx to model transitional menopause followed by surgical menopause, thereby removing residual hormones released from the follicle deplete ovary. **a)** After water radial-arm maze initial testing, a 4-hr delay between trials 2 and 3 was imposed. The graph depicts mean ± SE spatial working memory errors for each group for post-delay trials. The VCD-Ovx group made fewer errors relative to all groups, suggesting that transitional ovarian hormone loss before Ovx aids memory retention, and that removal of residual ovarian tissue after transitional ovarian hormone loss improves memory retention relative to retaining residual ovarian tissue, * p < 0.0005. **b)** Initial baseline performance on the delayed-match-to-sample spatial recent memory task. Trial 1 was the information trial, informing the animal where the platform was located on that test day. Shown are the mean±SE errors for each group for blocks 1 and 2 for the test trials. SHAM and VCD-Ovx groups made fewer errors during initial learning in block 1, relative to the VCD group, * p < 0.05, suggesting that transitional menopause without Ovx impaired performance, while Ovx initiated after the transition enhanced performance. The Ovx group was marginally different from the VCD group, # p = 0.06.

In the rodent, transitional hormone loss can be induced via the industrial chemical 4-vinylcyclohexene diepoxide (VCD), which produces follicular depletion by selectively destroying primordial and primary follicles via acceleration of the natural atresia process, resulting in hormone profiles more similar to naturally menopausal women versus Ovx [27,117,118]. Data from work using this novel menopause model, like data from work using the Ovx model, have indicated that ovarian hormone loss impacts cognition. We recently showed, using rats, that VCD-induced transitional menopause impaired learning of a spatial recent memory task, and that transitional menopause before Ovx was better for spatial memory than an abrupt loss of hormones via Ovx only with no transition (Figure 3) [87]. These results correspond with findings from Rocca and colleagues [119] showing women that had undergone oopherectomy prior to menopause onset had elevated cognitive impairment risk compared to age-matched women without oopherectomy. Collectively, the findings suggest that initiation of transitional menopause before surgical ovary removal might benefit mnemonic function and could obviate some negative cognitive consequences of surgical menopause. 

Future studies addressing this important question of cognitive effects of type of ovarian hormone loss, combined with evaluation of potential interactions between type of ovarian loss and subsequent hormone therapy, should yield valuable insights and new interpretations of variables impacting cognitive outcome due to ovarian hormone loss and therapy in menopausal women.

#### 3.5.2. Estrogens and cognition

Estrogens are a class of hormones including 17β-estradiol, estrone, and estriol; 17β-estradiol is the most potent naturally-circulating estrogen, followed by estrone and estriol, in order of receptor affinity [120,121]. 

To date, 17β-estradiol has been the primary estrogen used to test cognitive effects of hormone therapy in the animal model. The majority of studies evaluating activational effects of estrogens in an Ovx background for spatial learning and memory have been performed in young rodents, with many showing enhancements due to treatment [111,113,122,123,124,125,126,127,128,129,130,131,132]. In young and middle-aged rodents, one of the most consistently reported memory enhancements due to 17β-estradiol treatment is on the visual object recognition task whereby animals are tested on whether they “recognize” a novel object (operationally defined by increased exploration of the novel object) [e.g., 127,133,134]. Generally, this task is considered non-spatial and it is thought to be non-hippocampal dependent, although exceptions have been noted [135,136,137] and benefits of 17β-estradiol hippocampal infusions have been seen [138] thereby supporting a role for the hippocampus in this task. Nonetheless, the consistent findings in the literature on this acute task indicate that estrogens can rapidly enhance recognition memory. Similarly, studies in young Ovx monkeys have shown benefits due to 17β-estradiol treatment for certain measures such as visuospatial attention [139], but no benefits of 17β-estradiol treatment for other specific aspects of learning and memory [140,141] (the authors wish to note the distinction between the terms “estrogen”, “estradiol”, “17α-estradiol”, “17β-estradiol” and other estrogenic structures [142]. There are several estrogenic compounds, with differences in their molecular structure that may confer variable pharmacokinetic and pharmacodynamic profiles, which can all be termed “estrogens” when referring to them as a class. It is important to note research findings in the context of the specific estrogen used. The review herein is specific about the estrogen used in the literature when so noted in the original manuscript. If the specific estrogen is not noted in the original manuscript, the term used in the original work is also used to describe the findings here).

Recently, the context of studies experimentally evaluating effects of estrogens and progestins in aging animal models is usually to understand whether estrogen-containing hormone therapies impact cognition within this older cohort, so that findings might be eventually translated to optimize hormone therapies given to menopausal women (for examples see [103,143]). Since the first controlled clinical evaluation showing that 17β-estradiol injections given to 75 year-old women enhanced memory [144], there have been numerous studies showing cognitive decline after ovarian hormone loss, and enhancement after treatment with various types of preparations containing estrogens, in menopausal women (for a review see [67]). Premarin, the most commonly prescribed hormone therapy given to women [145], is conjugated equine estrogens (CEE) which contains the sulfates of at least ten estrogens, is over 50% estrone sulfate, 20-25% equilin sulfate, and has only trace amounts of 17β-estradiol; after metabolism, the resulting biologically active circulating hormones are primarily estrone and, after estrone’s conversion, 17β-estradiol, as well as equilin [121,146]. It is hypothesized that these three metabolites are primarily responsible for the estrogenic effects of CEE [121], although there are other estrogens and related metabolites present that could initiate effects on their own; these hormones include, but are not limited to equilin, Δ^8,9^ dehydroestrone, dihydroequilin-17β, and equilenin [120]. CEE-containing therapy improved memory via self-report [147], case studies [72] and randomized psychometric evaluations [148]. Yet, to the surprise of many in the field, findings evaluating global cognitive function in the large placebo-controlled WHI Memory Study (WHIMS), funded by the National Institutes of Health, showed an increase in probable dementia risk and no effect on mild cognitive impairment in women 65 years or older taking the combination therapy CEE + the synthetic progestin, medroxy progesterone acetate (MPA) [149]. CEE alone showed a non-significant increase in incidence of probable dementia and mild cognitive impairment [150,151]. An ancillary study to the WHI testing more specific cognitive functions, the WHI Study of Cognitive Aging (WHISCA), reported that CEE+MPA therapy had a negative effect on verbal memory and a trend for positive effects on figural memory in women 65 and over free of probable dementia [152]. Most recently, the WHIMS-MRI study found that CEE use with or without MPA was associated with small but measurable atrophy in the frontal cortex and hippocampus, brain regions important for cognition [153]. 

Thus, at the present time the studies in women have little consensus. New studies will be crucial to the understanding and clarification of the complex effects of ovarian hormone loss and hormone therapies given during aging. Something remarkable now is happening in the field of ovarian hormones, memory and aging. there is a revitalized focus on the complexity and intricacy of this tertiary system so that novel mechanisms and understandings of the findings in women can be detailed. Identifying the effects of the various administration parameters and components of hormone therapies, including detailed evaluations using basic science and system approaches, is the optimal approach to converge the many findings that appear contradictory. In fact, as new data emerge it may become clear that the cognitive effects of hormone therapy are not contradictory at all. Rather, they may be dependent on numerous variables not yet taken into account in many studies. One way to further our understanding of gonadal hormone effects on spatial cognition is by using animal models. For example, female animal models can provide insight into the cognitive effects of ovarian hormones, allowing evaluative changes in cognition due to ovarian hormone withdrawal and treatment, while enabling experimental control not possible in clinical research. 

With the above in mind, we now ask, “*Do aged animals show cognitive enhancements after treatment with estrogens*?” The answer to this question is vague, “*Yes, but…*” Indeed, effects depend on a multitude of factors. It has become clear that the cognitive effects of estrogens are rich with complexity, and that they have a multidimensional nature that we are just starting to understand. In addition to age, estrogenic effects appear to be influenced by innumerable factors including, but not limited to, timing of hormone administration relative to hormone loss, dose, mode of treatment, and whether progestins are given concurrently. Two of the most interesting questions driving much of the newer animal research in the field of estrogenic actions on brain health during aging have been spawned from the WHIMS findings. Women who participated in the WHIMS were between 65-79 years old, and many had experienced ovarian hormone deprivation for a substantial amount of time before receiving CEE-containing treatment [154]. This presents the intriguing question of whether the older age of the women, and/or whether the extended window of time from menopause (which occurs in the 50s, on average), impacted outcome of CEE treatment.

Data collected within the last few years indicate that whether cognitive benefits of estrogen therapy are realized is influenced by the delay between Ovx and hormone treatment, with limited benefits seen when there is an extended window between ovarian hormone loss and 17β-estradiol treatment. This has been seen in the rat on tests of spatial memory, whereby the Daniel laboratory found 17β-estradiol replacement initiated immediately after Ovx enhanced spatial memory performance in middle-aged rats, but imparted no benefit when given 5 months after Ovx [95]. These behavioral findings correspond with neurochemical data from the same laboratory showing that in both young and middle-aged rats, 17β-estradiol treatment given immediately after Ovx increased choline acetyltransferase (ChAT; see section 4.2) levels in the hippocampus, while this increase was not seen when initiated 5 months after Ovx; it is noteworthy that this pattern was opposite of that seen in prefrontal cortex [155]. There may also be a critical window for the well-established findings that 17β-estradiol regulates dendritic spines in the hippocampus [156], as we found that a 10 week delay after Ovx decreased the effectiveness of 17β-estradiol to increase CA1 apical spine density as compared to treatment given immediately in young rats [129]. In the non-human primate, the important question of a critical window for cognitive efficacy of estrogens has not yet been directly addressed, but there is evidence that the surgically menopausal non-human primate is still sensitive to estrogenic effects in old age even if there is a substantial window after ovarian hormone loss. Indeed, benefits due to treatment with estrogens were seen when treatment was initiated immediately [157,158], within 30 weeks [159], or even when given 10-16 years [160] after Ovx. However, it is noted that this latter study used a within-subjects repeated measures design of placebo or estrogen treatments, and effects were transiently expressed, somewhat limiting interpretation of a specific critical window question since there were windows of absence and exposure across the timeframes of the study. 

A number of studies evaluated estrogenic effects on spatial ability in middle-aged or older female rodents, most using 17β-estradiol [53,89,93,94,96,97,98,99,161,162], or the synthetic or semi-synthetic estradiol preparations ethinyl estradiol or estradiol cypionate, in monkeys [159,160]. In general, studies indicate that 17β-estradiol treatment can enhance performance on cognitive tests when treatment is initiated during middle-age or in old age, as seen in rodents [53,88,89,93,94,96,98] and non-human primates [158,159,160], although the effect may be transient in monkeys for visual recognition, with benefits seen 12 weeks following treatment initiation, but not another 12 weeks later when tested 24 weeks after treatment initiation [158]. A similar transient effect of ethinyl estradiol treatment was seen in surgically menopausal aged monkeys for spatial delayed recognition, a test that shows hippocampal-lesion [163] and age-related [164] impairments, with benefits seen for months 2-4, but not 6-8, of the study [160]. There is also new evidence from our laboratory that both tonic and cyclic Premarin (CEE), at doses relevant to what women take as hormone therapy, can enhance spatial memory and retention and protect against cholinergic challenge on spatial tasks in middle-aged Ovx rats [90,165].

**Figure 4:**
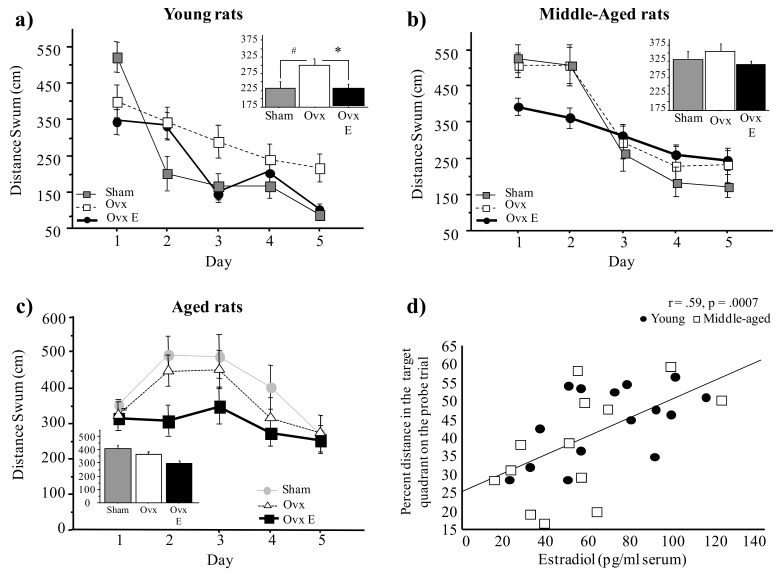
Age alters responsiveness to Ovx and 17β-estradiol treatment, and serum levels of 17β-estradiol correlate with memory scores in young and middle-aged rats [53]. Mean ± SE distance scores (cm) on the reference memory Morris maze for **a)** young Sham, Ovx and OvxE groups, **b)** middle-aged Sham, Ovx and OvxE groups, and **c)** aged Sham, Ovx, and OvxE groups; the inset graphs represent data collapsed across days. Ovx impaired performance on the latter portion of testing for young animals, while there was no effect of Ovx in middle-aged animals. 17β-estradiol treatment enhanced performance in both young and middle-aged groups. For aged Sham, Ovx, and OvxE groups there were no significant main effects or interactions for distance scores due to Ovx or 17β-estradiol replacement. Thus, there was a dissociation between sensitivity to Ovx and responsiveness to estrogen treatment. * p < 0.05, # p = 0.06, *** p < 0.0001. **d)** For the scatterplot, young animals are represented by filled circles, and middle-aged animals by unfilled squares. Higher circulating 17β-estradiol levels were correlated with better spatial reference memory, as tested on the Morris maze.

Age-related changes in responsiveness to treatment with estrogens have been shown for spatial cognition in studies directly testing multiple ages after 17β-estradiol, as compared to vehicle-control, treatment. Young and middle-aged Ovx rats showed a positive response to 17β-estradiol treatment on the spatial reference memory Morris maze task, and higher circulating 17β-estradiol levels correlated with better performance (Figure 4) [53]. Aged Ovx rats were not responsive to the 17β-estradiol treatment regimen that effectively enhanced spatial reference memory in young and middle-aged Ovx rats (Figure 4) [53], concurring with age-related interactions with 17β-estradiol replacement for spatial memory shown by others [96]. Why, then, have some studies shown that aged female rodents can exhibit cognitive enhancements in response to 17β-estradiol treatment? For example, 17β-estradiol injections enhanced spatial reference memory in 27-28 month old ovary-intact mice [94]. The difference in results may relate to type of 17β-estradiol administration as cyclic versus tonic, as priming with cyclic 17β-estradiol enhances responsiveness to tonic 17β-estradiol in older Ovx rats [98]. In the last few years, it has also become evident that issues associated with the experimental study designs themselves can affect outcome of gonadal hormone effects on cognition. For example, several minutes of handling obviated the spatial memory benefits seen with 17β-estradiol in Ovx rats [166], and experience with spatial maze testing obviated the increase in hippocampal dendritic spines normally seen after 17β-estradiol treatment in Ovx rats [167].

Whether 17β-estradiol replacement improves performance of aged animals may also relate to memory type and demand. Working memory enhancements due to tonic 17β-estradiol treatment have been reported in aged Ovx rats, although this effect is more pronounced when memory demand is high [93,161]. We and others have shown 17β-estradiol-induced spatial working memory improvements in young Ovx rats as well, an effect most pronounced when spatial working memory demand is high [111] and an effect that depends on administered 17β-estradiol dose [111,122,125,131]. Indeed, a higher supraphysiological 17β-estradiol dose may be necessary to enhance spatial reference memory retention in rats approaching old age [96]. These findings correspond with data from our laboratory showing that higher serum levels of exogenous 17β-estradiol treatment correlates with better spatial reference memory performance in young and middle-aged Ovx rats (Figure 4) [53]. It also appears that sensitivity and responsiveness to ovarian hormone loss does not predict sensitivity and responsiveness to 17β-estradiol treatment. For example, we found that young animals were responsive to both ovarian hormone removal and replacement, middle-aged animals were not responsive to ovarian hormone removal but were responsive to estrogen replacement, and aged animals were not responsive to ovarian hormone removal or replacement for spatial reference memory Morris maze performance [53].

Collectively, the data indicate that cognitive enhancements in aged females could occur due to treatment with estrogens if parameters are optimized. This may include a regimen of estrogen-including hormone therapy whereby there would be a short, limited time window between ovarian hormone loss and estrogen treatment, initiation of treatment would occur earlier in life [in the early phase of menopause (e.g., [168]), and benefits would likely be most evident in times of high memory demand. It should be noted that currently, in their 2010 position statement on estrogen and progestogen use in postmenopausal women, the North American Menopause Society states that hormone therapy “cannot be recommended at any age for the sole or primary indication of preventing cognitive aging or dementia” since hormone therapy increased dementia incidence in women 65 years of age and older in the WHIMS [169]. To follow is the statement that “available data do not address whether hormone therapy used soon after menopause increases or decreases later dementia risk.” Future studies, spanning basic science to clinical, are clearly warranted in this regard. As well, since research is revealing new mechanisms of estrogenic effects on cognition (see section 4, below), novel therapeutics combining pharmaceuticals and commonly-used hormone therapies will likely provide novel hormone therapy options for women, and should be strongly pursued as future lines of investigation [170,171].

#### 3.5.3. Progestins and cognition

Estrogens’ effects on cognition can be impacted by the presence of progesterone, suggesting, again, that gonadal hormone effects on cognition are multidimensional and complex. 

**Figure 5:**
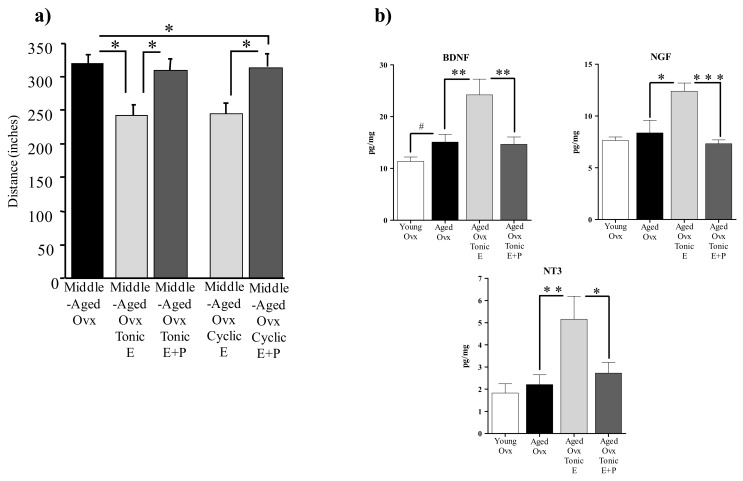
Progesterone reverses 17β-estradiol’s effects on spatial memory and neurotrophins [97,184]. **a)** Mean ± SE distance-to-platform (inches) on the Morris maze task for each treatment group. Tonic and cyclic 17β-estradiol treatments enhanced spatial reference memory performance in middle-aged Ovx rats, while the addition of progesterone reversed these beneficial cognitive effects. *p < 0.05. **b)** Mean ± SE protein levels of BDNF, NGF, and NT3 in entorhinal cortex for aged Ovx rats receiving no hormone (Aged Ovx), a 17β-estradiol pellet (Aged Ovx E), a 17β-estradiol pellet plus two progesterone pellets (Aged Ovx EP), and a young group also receiving Ovx surgery, with no hormone treatment (Young Ovx). Findings suggest that for all three neurotrophins, 17β-estradiol increased protein levels, while the addition of progesterone reversed these increases.# p < 0.07; * p < 0.05; ** p < 0.01; *** p < 0.0001.

Discussion regarding, and research testing, the combined regimen of estrogens plus progestins has increased as of late. This has been mostly due to hopes in understanding the findings that menopausal women taking CEE alone did not differ significantly from those taking placebo for dementia diagnoses [151], while, in contrast, twice as many women receiving CEE plus the synthetic progestin MPA were diagnosed with dementia as compared to the placebo group, a significant effect [149]. Of note, women with a uterus who are taking estrogens must include progestin in their regimen because of the increased risk of endometrial hyperplasia associated with unopposed estrogen treatment [172]. We have shown that progesterone abolished 17β-estradiol-induced benefits on the spatial reference memory Morris maze in middle-aged rats (Figure 5a) [97]. Accordingly, progesterone plus 17β-estradiol injections impaired performance on the spatial reference memory Morris water maze, while 17β-estradiol or progesterone treatment alone did not influence performance; this pattern of effects was not seen on the non-spatial Morris maze suggesting this combination treatment has specific effects within the spatial domain [173]. These effects may not translate to working memory tasks, however, as progesterone treatment enhanced 17β-estradiol’s effects on a delayed-match-to-position spatial t-maze [93].

Progesterone alone has been associated with detrimental cognitive effects, in both clinical and preclinical studies. The “maternal amnesia” phenomenon in pregnant women is hypothesized to result from high circulating progesterone levels during late pregnancy [174]. In healthy women, a large oral progesterone dose is detrimental to memory [175]. High circulating progesterone levels are also observed in most rats following estropause [176], and many endocrine studies have shown that aged female rats enter a pseudopregnantestropause state, whereby progesterone values become significantly elevated but 17β-estradiol levels remain relatively unchanged [177,178]. This pseudopregnant state has been associated with poorer spatial cognition [179]. Correspondingly, one study has shown that in young, cycling rats spatial maze performance was worse during the proestrus phase, when estrogen and progesterone levels are at their highest, and best during the estrus phase, when estrogen and progesterone are at their lowest [180][but see 181,182]. 

**Figure 6:**
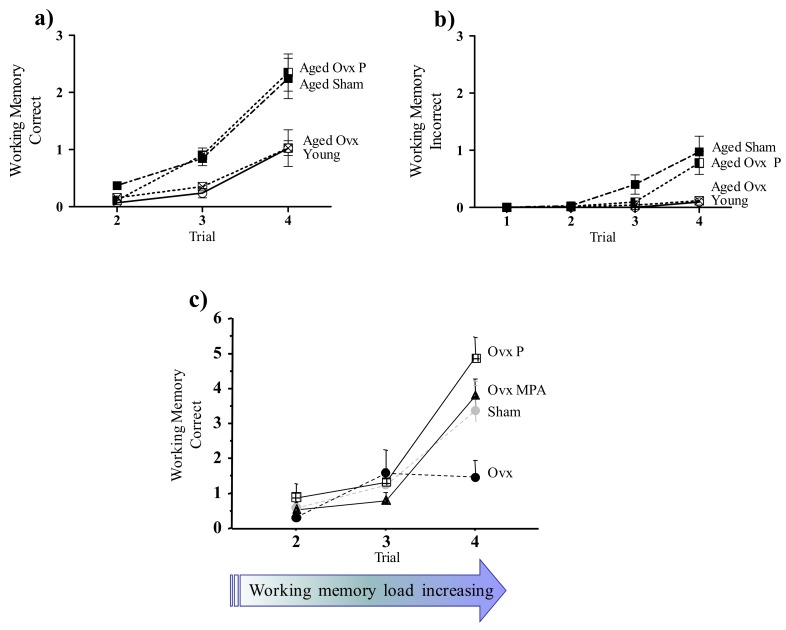
Progesterone reverses the cognitive enhancing effects of Ovx in aged animals, an effect paralleled by the synthetic progestin medroxy progesterone acetate (MPA) [58,91]. **a)** and **b)** represent the mean ± SE number of working memory correct and incorrect errors, respectively, committed on the water radial-arm maze for Young, Aged Sham, Aged Ovx, and Aged Ovx P (progesterone) treated animals [for error definitions see 58]. Young Sham and Aged Ovx rats were better able to sustain successful performance as trials and working memory load increased, compared to Aged Sham and Aged Ovx rats treated with progesterone (Aged Ovx P). Thus, on the two orthogonal spatial working memory measures, aged animals showed impaired performance as memory load increased relative to young animals, and Ovx enhanced performance in aged rats. Progesterone abolished the Ovx-induced enhancement. **c)** represents the working memory load effect whereby Sham, OvxP, and OvxMPA animals exhibited more mean±SE errors than Ovx animals on the last trial, indicating that animals treated with progesterone or MPA were impaired as the demand on working memory increased. This replicated prior findings that Ovx enhanced, and progesterone impaired, performance as memory load increased, and showed that the synthetic progestin, MPA, impaired spatial working memory as memory load increased in aged surgically menopausal rats.

Ovx in aged rats improves cognition [57], which is likely related to the removal of elevated progesterone levels seen with estropause. Indeed, progesterone administration has large detriments on spatial working and reference memory and reverses the beneficial effects of Ovx seen in aged rats (Figure 6 a & b) [58,91]. We recently extended these findings using the synthetic progestin MPA, which is the progestin component in the commonly used hormone therapy Prempro. MPA impaired spatial memory retention and exacerbated overnight forgetting on the spatial reference memory Morris maze [91]. Effects were also seen for working memory. Noting the same pattern seen with detrimental effects of aging, and beneficial effects of 17β-estradiol treatment, progesterone and MPA supplementation had the most marked performance effects on the water radial-arm maze at the highest working memory load, with progestin-treated aged Ovx rats showing disproportional impairments as working memory load reached its highest demand (Figure 6c) [58,91]. 

The collected findings indicate that progestins have a negative consequence on neuronal health, yielding detrimental effects on brain functions such as learning and memory. This is exemplified across multiple realms of the literature, from clinical to basic science. As discussed above, clinical evidence shows detrimental cognitive effects of combined hormone therapy [149] and basic science evidence shows that both MPA [91] and progesterone [58,91] impair cognition, and that progesterone abolishes 17β-estradiol-induced memory enhancements (Figure 5a) (but see [93,97,183]) and attenuates 17β-estradiol’s neurotrophic effects *in vivo* (Figure 5b) [184] and in cell culture [185]. Moreover, when comparing MPA and progesterone, it is noteworthy that MPA does not demonstrate neuroprotective properties, while progesterone does [186], and that MPA causes a greater attenuation of 17β-estradiol’s neurotrophic actions [186]. This *in vitro* work is in accordance with our laboratory’s recent findings that MPA has broader detrimental effects for learning and memory as compared to progesterone. In fact, progesterone’s detrimental effect on learning seems to be more of a transient nature [91]. Furthermore, while no study has directly evaluated the impact of MPA on cognitive health in younger women using this pharmaceutical as a contraceptive, it should be noted that these effects could translate to cognitive impairments in these younger women as well and account for some of the negative effects reported with use of this drug. Indeed, a documented case study reports amnesic effects corresponding with use of DepoProvera, a contraceptive wherein MPA is the sole hormone component [187]. The accumulating evidence therefore indicates that while a progestin is necessary as part of hormone therapy in women that have a uterus [172,188], the use of exogenous progestins does not result in a positive impact on brain health and function during aging in females. Some exciting future avenues might be brain specific estrogens [189], which would obviate the need for a progestin since estrogen-induced uterine stimulation from the hormone therapy would not occur.

### 3.6. Impact of androgens on cognition

Similar to the postulated relationship between estrogen loss and memory decline in aging women [109], recent studies suggest that a decline in testosterone levels is related to age-associated memory changes in men [190]. In men, testosterone levels decline slowly with age, and they decrease to only about 40% lower than levels seen in younger men, as compared to the more drastic loss of estrogens and progesterone seen in women after menopause [191,192]. One of the most striking relationships regarding this research area was discovered in the last decade, with reports that lower testosterone levels are linked with a higher risk of AD [193,194,195,196]; for example, lower serum testosterone levels are seen in male AD patients versus controls [193]. This work spawned many hypothesis-driven studies targeting the question of whether testosterone is linked to cognition in neurodegenerative disease and normal aging in general, and whether testosterone could enhance cognition in AD patients or in individuals with normal age-associated memory impairment. 

Relationships between endogenous testosterone levels and cognition have been observed in younger and older individuals, with, in general, the strongest relationships seen in the older population in retrospective and randomized treatment studies. Testosterone was related to cognitive performance in young men and women, and spatial ability was related to the change in seasons in accordance with seasonal alterations in testosterone levels [197,198,199]. Retrospective studies have suggested there is a relationship between a greater age-related cognitive decline and lower bioavailable testosterone levels [194,200,201]. In general, in older men, higher endogenous testosterone levels have been linked to better cognitive function, while an inverted U-shaped dose-response relationship between circulating testosterone and cognition has also been noted, with most beneficial effects with moderate circulating testosterone levels [200,201,202]. One of the most convincing pieces of evidence that testosterone effects on cognition hold to an inverted U-shape function for older men was recently reported. This randomized, placebo-controlled study evaluating healthy older men found that weekly testosterone injections resulting in moderate increases in serum testosterone or its metabolites yielded enhanced spatial and verbal memory, while the testosterone injections resulting in relatively smaller, or relatively larger, testosterone increases yielded no significant change [203]. Of note, the moderate increases due to the testosterone treatment that enhanced memory resulted in circulating levels that were normal to high-normal levels seen in young men, and the large increases due to the testosterone treatment that did not enhance memory were pushed into the supraphysiological range. Work evaluating endogenous levels also suggest a non-linear U-shaped function for testosterone levels and spatial ability with moderate levels optimal. For example, lower relative levels of salivary testosterone in men, and higher relative levels of salivary testosterone in women, were related to higher spatial ability scores [202]. It is possible that accumulating data will continue to support this inverted U-shaped quadratic relationship, and will help to synthesize the range of findings including, for example, newer work showing that age-related declines in endogenous testosterone levels did not directly correlate with age-related cognitive declines in spatial abilities, such as that seen on the mental rotation test [204]. As well, the first study testing the effects of testosterone supplementation in monkeys was published in the last year, and found that supraphysiological testosterone levels did not yield benefits on spatial cognition in young male monkeys; whether lower physiological levels within the postulated optimal range of the inverted U-function would have yielded benefits is yet to determined [205]. 

Placebo-controlled testosterone treatment studies have reported enhanced spatial cognition in healthy older men [203,206,207,208], an effect not seen in younger men [209]. Men with AD or mild cognitive impairment who received testosterone supplementation showed improved spatial cognition [210,211], although benefits of testosterone replacement were not seen in a pilot study evaluating old men with early- to mild- cognitive impairment and pre-treatment low levels of bioavailable testosterone [212].

**Figure 7:**
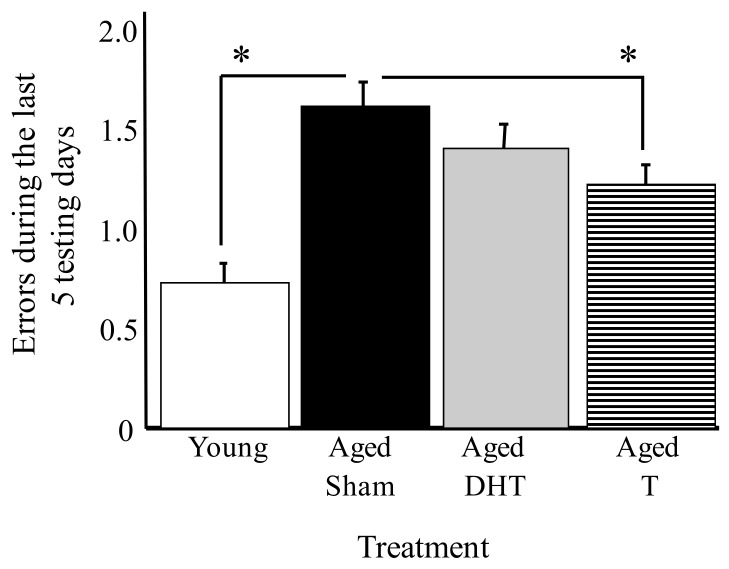
Testosterone supplementation decreased spatial working memory errors in aged male gonadally intact rats [215]. Rats were not castrated in this study in an attempt to conserve the hypothalamic/pituitary/gonadal axis, as this axis is typically conserved in older men. The administered androgen treatments were thus supplemental therapies. The aged male rats were divided into three treatment groups (n = 11 per group): sham (Aged Sham), dihydrotestosterone (Aged DHT), testosterone (Aged T). There was also a young male group which received sham surgery. Aged sham rats made more mean ± SE spatial working memory errors than young sham rats, and aged rats treated with testosterone (Aged T) committed fewer spatial working memory errors than aged rats receiving vehicle treatment (Aged Sham). * *p* < 0.05, *** *p* < 0.0005

Since testosterone can be converted to either dihydrotestosterone (DHT), which binds to androgen receptors, or to estrogen via the aromatase enzyme, testosterone’s mnemonic effects could be due to either DHT or conversion into estrogen [213]. In fact, 80% of circulating estradiol is not of testicular origin in men; it is from aromatization of testosterone occurring in the periphery or brain areas, including the hippocampus [213,214]. However, recently, an elegant study by Cherrier and colleagues showed that the spatial memory benefits of testosterone were seen in men even when aromatization to 17β-estradiol was pharmacologically blocked, and blood levels confirmed that the testosterone plus aromatase inhibitor reduced 17β-estradiol levels by 50%, thereby indicating the testosterone-induced spatial memory improvements occurred in the absence of concomitant 17β-estradiol increases [210]. However, this does not preclude interactions between testosterone and estrogens for spatial cognitive performance. We have shown that testosterone supplementation given to gonad-intact aged male rats enhanced learning on a spatial working and reference memory task, and improved spatial working memory[215]. These effects may be due to an interaction between testosterone and estrogens; indeed, we found that DHT, which is not converted to estrogens, had no effect on spatial maze scores in aged male rats (Figure 7) [215]. Accordingly, others have shown enhanced spatial memory retention after testosterone, but not DHT, treatment in aged mice [216]. Of note, in the Bimonte-Nelson *et al.* study [215], the vehicle-treated gonad-intact aged sham rats exhibited relatively higher serum 17β-estradiol levels and showed compromised maze performance. Conversely, testosterone treatment decreased serum 17β-estradiol levels and improved maze performance, compared to the gonad-intact aged sham rats. These combined findings suggest that testosterone did not initiate its effects by increasing serum 17β-estradiol levels, and that the group of aged rats that exhibited the best performance, the testosterone treated group, showed relatively higher testosterone and lower 17β-estradiol circulating levels. Similarly, in older men, better cognitive performance was related to higher circulating testosterone and lower 17β-estradiol levels [200] and testosterone treatment improved spatial cognition in older men, while at the same time it decreased 17β-estradiol levels [208]. It is clear that testosterone can impact spatial cognition, and that these effects may involve estrogenic interactions. 

There is accumulating evidence that the androgen and rostenedione may be involved in cognition. In VCD-treated female rodents, similar to menopausal women the ovarian endocrine milieu becomes androgen rich after follicular depletion [27,118]. Using VCD-induced transitional menopausal rats our laboratory recently found that higher circulating levels of and rostenedione were associated with impaired working memory performance [87], a correlation later replicated in our laboratory on two orthogonal measures of working memory as well as reference memory [92]. Importantly, it is noted that these findings are the only evidence thus far demonstrating that and rostenedione impacts cognition, and these findings are correlative. Future methodical experimental evaluation of and rostenedione is warranted, including direct administration to evaluate if impairments are induced, and therapeutic blockade to determine if previously observed impairments are attenuated. 

### 3.7. Impact of gonadotropins on cognition

While it is well established that the gonadotropins follicle stimulating hormone (FSH) and lutenizing hormone (LH) are involved in regulating reproductive functions via negative and positive feedback loops, it is becoming increasingly apparent that gonadotropins might, directly or indirectly, impact cognitive function as well, including within the spatial domain. Receptors for both LH [217] and FSH [218] are found in the rat hippocampus. Although there have been few links between FSH and cognition [e.g., 65,87], there is strong evidence that LH is related to cognition, with perhaps the strongest evidence from the aging and neurodegenerative disease literature [219]. For example, in aged men, higher LH levels were associated with poorer performance on an immediate recall task [220,221]. Supporting plausibility of LH effects on spatial cognition, the highest density of LH receptors in the brain is found in the hippocampus [222,223], and LH-releasing hormone has been found in a neuronal-derived septal cell line with cholinergic properties [224], suggesting the presence of LH effects in regions intimately involved in spatial learning and memory which are also affected by aging and AD. 

**Figure 8:**
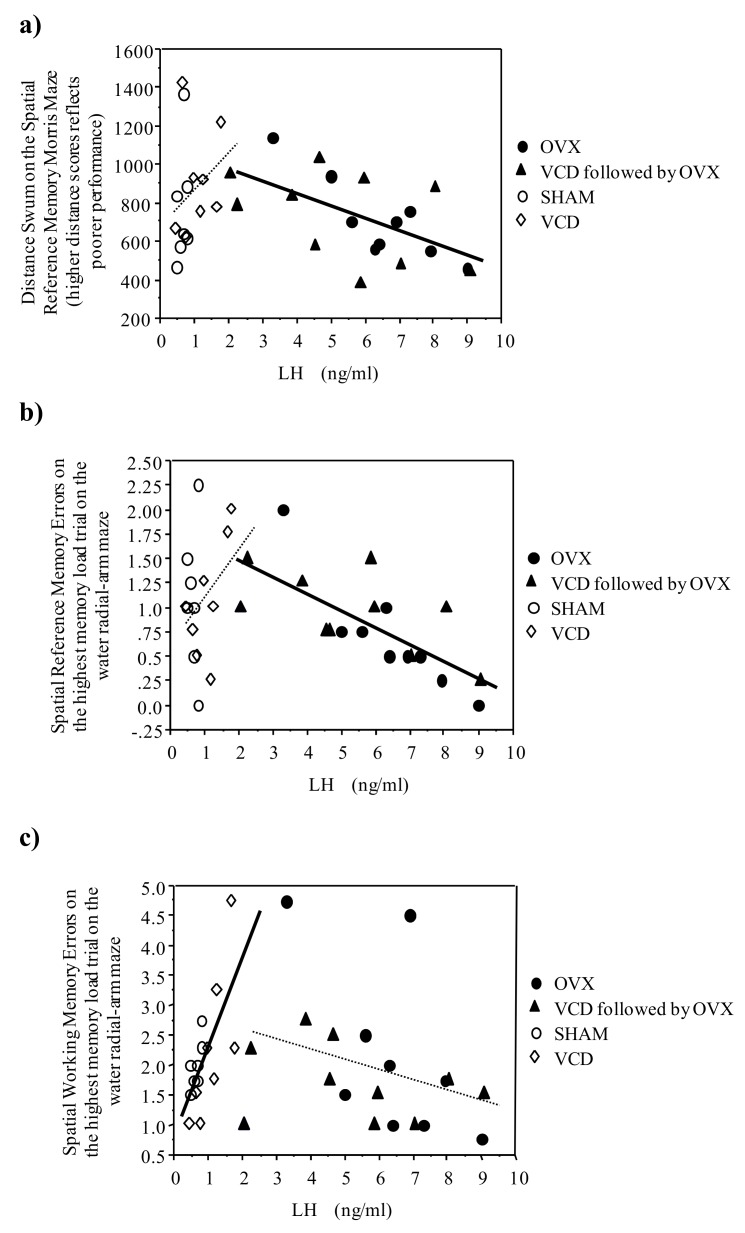
Circulating LH levels show a curvilinear relationship with memory scores [87]. Regression analysis indicated that in animals without ovaries (Ovx and VCD-Ovx), higher LH was associated with better reference memory for the a) Morris maze and, b) water radial-arm maze. As shown in c), in animals with ovaries (SHAM and VCD), higher LH was associated with worse working memory; indeed, higher LH was associated with more water radial-arm maze working memory errors. Graphically, the significant regression coefficients for these analyses are shown as solid lines; the dashed lines of the other groups are shown for comparison purposes to aid interpretation.

In a recent study by our laboratory evaluating VCD-induced follicular depletion and Ovx effects on cognition in the middle-aged rat model, there was a clear inverted U-shaped function for serum LH and number of spatial memory errors. We found that the highest and lowest LH levels were associated with the best performance, an effect not seen with FSH [87]. This relationship with LH became apparent in scatterplots including all treatment groups so that the range of values across groups could be noted. Indeed, this range was broad because, as expected, Ovx increased LH levels due to lack of ovarian hormone negative feedback after ovarian hormone loss, while Sham Control animals showed LH values in the relatively lower range. When LH levels ranged from approximately 0-to-2 ng/mL, higher LH levels correlated with worse maze performance to reveal a positive relationship with errors. However, when LH levels ranged from approximately 2-to-10 ng/mL, higher LH levels correlated with better maze performance to reveal a negative relationship with errors. When the groups were put in the same scatterplot the effect was a striking inverted U-shaped function (Figure 8). This pattern was seen for multiple measures, including spatial working and reference memory. While limitations exist in interpreting this relationship between LH and memory scores in this study because LH levels were confounded by group membership, this quadratic relationship is nonetheless striking, especially given the increasing evidence that LH levels are linked to cognition and pathologies associated with neurodegenerative disorders [219,221]. Other studies report higher LH levels are related to better cognitive performance, similar to the effects seen in our Ovx animals. Tonic treatment with LH-releasing hormone, elevating LH concentrations to Ovx levels, enhanced performance on visual-discrimination in young rats [225], and enhanced non-spatial working memory in aged rats [226].That higher LH levels were associated with better memory in these studies is likely related to LH levels being increased to that of Ovx animals. On the other hand, corresponding with our Acosta *et al.* [87] findings in ovary-intact animals that higher LH levels correlated with worse cognitive performance, in ovary-intact aged female mice, experimentally-induced LH reductions decreased amyloid-β concentrations and enhanced cognition, while LH increases promoted biochemical brain changes consistent with AD, although none of these studies correlated circulated LH levels with memory scores in individual animals [227,228,229]. Also, men and women with AD had higher circulating LH levels than controls [227,230].

Thus, over the last decade there has been increasing evidence that LH levels are related to cognition and possibly pathologies associated with neurodegenerative diseases such as AD. It is unclear whether these cognitive effects are directly due to fluctuations in LH levels or whether they are indirectly related as a consequence of ovarian hormone alterations. The growing literature evaluating the relationship between LH and cognition suggests that it may subserve an inverted U-shaped function, with an intermediate level resulting in optimal brain function. This is an important area that will require further study, with results possibly revealing important mediators of cognitive function. 

## 4. Gonadal Hormones, Spatial Cognition and Aging: Postulated Brain Mechanisms

Understanding the brain mechanisms of how gonadal hormones and aging impact spatial cognition is key to unlocking the mysteries of this complex relationship and will likely help lead the path to new therapeutics to aid cognitive aging. There is accumulating evidence that gonadal hormones affect multiple brain parameters related to cognitive functioning, that aging affects multiple brain parameters related to cognitive functioning, and that gonadal hormones impact these parameters differently depending on age. These brain changes almost certainly encompass multiple brain regions and domains, and span innumerable variables [231,232,233,234], only some of which we describe below. Indeed, to follow is a discussion of what we hypothesize is the most likely candidates relating to mechanisms underlying gonadal hormones’ impact on cognitive aging.

### 4.1. Synaptic plasticity in cognitive brain regions

Brody reported in 1955, from counting neurons in human brains from people aged birth to 95, that decreases in cortical neuron number partially accounted for the age-related reduction observed in brain weight [235]. Early hypotheses related to this finding, and asserted that functional changes in the brain during normal aging were associated with substantial neuron loss including in cognitive brain regions such as the hippocampus, subiculum, and prefrontal cortex [236,237]. Recent unbiased stereological studies indicate that, on the contrary, widespread loss of neurons does not occur during normal aging, at least in most brain areas (discussed in [12]). One notable exception to this was reported in 2004; Smith and colleagues found a selective and focal reduction of neuron number, as determined by stereology, in dorsolateral prefrontal cortex region 8A in aged cognitively-impaired non-human primates [238]. Remarkably, this specific prefrontal region has been previously associated with working memory performance on the task that the evaluated non-human primates showed impairment [239,240,241,242,243]. Aside from this research, the majority of the literature indicates that neuron number is largely preserved as aging ensues. One of the most elegantly performed studies on this question to date found that total neuron number in the parahippocampal region (entorhinal, perirhinal and postrhinal cortices) was stable during normal aging in the rat, as quantified by contemporary stereological techniques [244]. In fact, even though in this study there was individual variability in spatial learning among the aged rats, this performance variability failed to correlate with the number of neurons in any evaluated region and there did not appear to be more loss in the aged animals with the most severe cognitive impairments. While the general consensus in the literature is that age-associated changes in cognition can occur in the absence of a substantial or significant widespread decrease in total neuron number, accumulating evidence supports the assertion that aspects of neuron structure *are* compromised as aging ensues, and that age-related cognitive changes involve alterations in connectivity.

The mammalian brain shows considerable plasticity, especially in regions related to learning and memory such as the hippocampus and neocortex. Indeed, the hippocampus contains one of the rare sites for neurogenesis that persists long after early development [245,246,247]. Age-related cognitive decline likely involves neoplastic changes within the brain [12], such as alterations in dendritic structure, spine number, and spine shape. Using various techniques, there is evidence that hippocampal and neocortical synapses decrease with normal aging [248]. Moreover, there are alterations in factors influencing synaptic transmission that occur with normal aging, including the reductions mentioned above in synaptic density as well as dendritic regression in apical and basal neuron regions in brain areas established as mediators of memory processing [249]. As technology advances, scientists can use these techniques to decipher the details of these changes and their relation with cognitive aging. To follow, the tremendous complexity of the aging brain and brain-memory interactions will be better understood. For example, a recent study evaluating non-human primates found age-related decreases in spine density as well as dendritic diameter, length and branching complexity in apical dendrites of “long” projection neurons from superior temporal cortex to the prefrontal cortex [13]. However, in “local” projection neurons within the prefrontal cortex, only spine parameters were reduced [13]. These findings are especially exciting because evaluations were conducted on pyramidal neurons known to be important in working memory circuits, in a brain region intimately linked to memory processing [250,251,252,253]. This research is a superb example of how knowledge and discoveries of subtle morphological changes can yield insight into complexities of brain dysfunction during aging. Combining such work with hormone status will undoubtedly yield exciting revelations of hormone, memory, and aging interactions.

Gonadal hormones, especially estrogens, have been demonstrated to have significant effects on neuron morphology (for a review see [254]). Estrogens were first shown to influence brain regions historically known for roles in reproduction, such as the hypothalamus, in rodents [255,256,257]. Other early work found that hormone fluctuations with the estrous cycle affect regions involved in cognition [258,259]. Subsequent studies extended these findings to show that estrogens alter neuron morphology by increasing hippocampal CA1 spine density in Ovx rats [260,261,262,263], and that testosterone elevates hippocampal CA1 spine density in Gdx male rats [264]. The testosterone effect was apparently not due to testosterone’s conversion to 17β-estradiol, as in this same study 17β-estradiol had no effect on CA1 spines, while DHT did [264]. Dendritic spines have long been theorized to be a structural component of memory [265,266,267,268,269], leading to wide-reaching implications regarding estrogen’s and androgen’s influence on cognitive function. This tenet is supported by recent work showing that spatial working and reference memory performance is enhanced after 17β-estradiol injections [129,131,270] within the timeframes corresponding to 17β-estradiol-induced increases in hippocampal dendritic spines [260,261,262,263] and other markers of synaptic plasticity [259]. Providing more direct support of the link between ovarian hormones, memory and spines, Ovx in rats impaired object recognition and place recognition memory, and significantly decreased spine densities in pyramidal neurons of the medial prefrontal cortex and the CA1 region of the hippocampus [116].

While differences exist in the cortication between the human, non-human primate, and rodent brain that lead to functional differences between the species, research has shown that the frontal and prefrontal cortices in all three species are involved in working memory processing even though the rodent regions may not be entirely homologous to the primate [250,251,252,253,271]. Numerous rodent studies have demonstrated working memory enhancements after 17β-estradiol treatment [95,111,122,272,273,274]. Similarly, 17β-estradiol with and without progesterone enhanced working memory in young and aged non-human primates [158,159,160], and working memory varied across the menstrual cycle in young monkeys [275]. Although it is important to note that null working memory findings have also been reported after Ovx and 17β-estradiol treatment in non-human primates, which may be related to task demand (*i.e.,* only visual vs. spatial working memory) [140,158]. Similar to findings in the hippocampus [276], 17β-estradiol alters the number of prefrontal cortex dendritic spines. Short-term 17β-estradiol administration increased the number of dendritic spines in the prefrontal cortex of non-human primates [277], an effect also found after long-term treatment [278]. Taken together, it is conceivable that 17β-estradiol working memory enhancements are concomitant with increases in prefrontal cortex spine number. In fact, 17β-estradiol may facilitate working memory by affecting prefrontal neuronal excitability and subsequent synaptic plasticity, similar to mechanisms seen in the hippocampus, although further research is necessary to confirm this link. 

### 4.2. Basal forebrain cholinergic neurons

**Figure 9:**
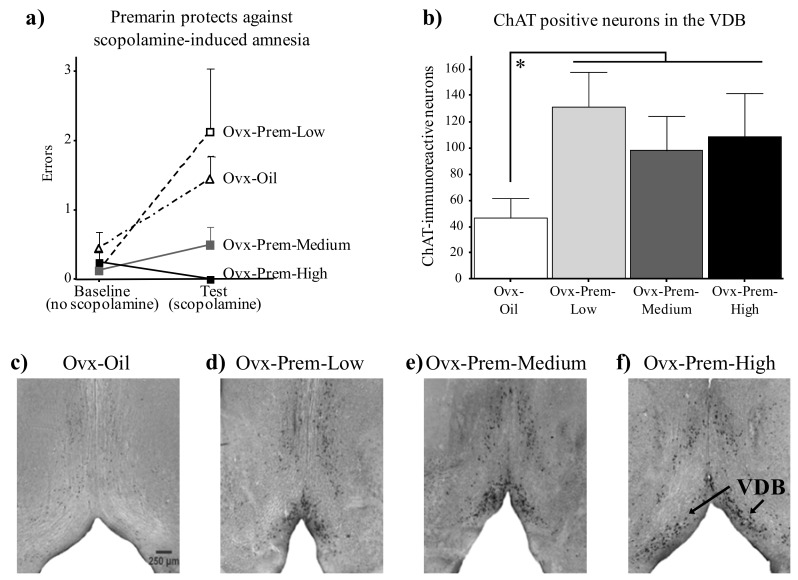
Premarin (CEE) treatment protects against scopolamine-induced amnesia and increases the number of ChAT positive neurons in the VDB of the basal forebrain in middle-aged Ovx rats [165]. Middle-aged Ovx rats received either chronic cyclic treatment with vehicle injection (sesame oil, Ovx-Oil) or one of the following amounts of injectable Premarin (as prescribed to women): 10 (Ovx-Prem-Low), 20 (Ovx-Prem-Medium) or 30 μg (Ovx-Prem-High). **a)** Mean ± SE total number of errors for each treatment group after scopolamine (0.2 mg/kg, IP) challenge. Premarin dose-dependently protected against scopolamine-induced amnesia on the delayed-match-to-sample working memory plus maze. **b)** Mean ± SE ChAT immunohistochemically stained cells were quantified in the basal forebrain in behaviorally tested animals. All Premarin-treated groups were collapsed and compared to the Ovx-Oil group. In the VDB, Premarin-treated animals had more ChAT-immunoreactive neurons relative to the Ovx-Oil group, ps< 0.05. Photomicrographs showing ChAT-immunoreactive neurons in sections through the BF from animals in the **c)** Ovx-Oil, **d)** Ovx-Prem-Low, **e)** Ovx-Prem-Medium, **f)** Ovx-Prem-High.

One of the most convincing and studied neurobiological links between gonadal hormones and memory is the work assessing function and viability of the cholinergic system, with an emphasis on basal forebrain cholinergic neurons. Abundant evidence suggests that estrogen-induced effects on learning and memory are mediated, in part, via the cholinergic system (for review see [171]). For example, studies directly testing the link between cholinergic neurons, neurochemistry, and memory performance have shown that administration of 17β-estradiol potentiated increases in hippocampal acetylcholine levels during maze learning relative to vehicle treatment in Ovx animals [128], and that 17β-estradiol-induced memory improvements were present in animals with intact basal forebrain cholinergic neurons, but not in animals with basal forebrain cholinergic lesions [279,280]. Studies have also shown that 17β-estradiol treatment alters multiple markers of ChAT in middle-aged and aged animals, which appears to depend on duration of ovarian hormone deprivation before treatment, as well as specific brain region and basal forebrain nuclei evaluated [155,281,282]. In young adult Ovx rats, delayed-match-to-position acquisition correlated with increased ChAT activity in two targets of basal forebrain cholinergic innervation, the hippocampus and frontal cortex [279], and high affinity choline uptake was increased in these regions [132,283]. There is additional direct evidence that basal forebrain cholinergic neurons, and their projections to hippocampus and frontal cortex, are involved in estrogen’s cognitive effects. 17β-estradiol administration increased ChAT levels in the horizontal limb [281,284,285] and in projection sites to hippocampus and cortex [114,132,285], as well as restored basal forebrain ChAT mRNA expression [286,287]. Notably, there are also studies showing that 17β-estradiol increased the number of ChAT-immunoreactive (IR) neurons in the medial septum (MS) and the vertical diagonal band (VDB) of the basal forebrain in young Ovx rats [288,289], and increased the number and size of MS ChAT-IR neurons in middle-aged Ts65Dn mice, a mouse model of Down syndrome [290]. In aged monkeys 17β-estradiol treatment increased the number of ChAT-IR neurons in the VDB [282]. Recently, we found that Premarin treatment influenced the cholinergic system. Premarin prevented the amnestic effects of the cholinergic muscarinic antagonist scopolamine as well as increased the number of ChAT-IR neurons in the VDB of middle-aged Ovx rats (Figure 9) [165], similar to effects seen with 17β-estradiol in young rats [288,289]. 

In specific regions within the rat basal forebrain, ChATis influenced by natural hormone fluctuations across the estrus cycle [291], which may be related to cognitive findings noted by others in the rat [180]. Additional work has shown that ChAT markers are impacted by the specific dose and duration of hormone treatment [289,291]. The majority of the groundbreaking work defining ovarian hormone effects on basal forebrain cholinergic neurons has been done in Gibbs’ laboratory. For example, in 1996 Gibbs showed that in cycling rats, the highest levels of ChAT mRNA in the MS occurred during diestrus 1, while the highest levels during diestrus 2 were detected in the nucleus basalismagnocellularis (NBM), indicating important region-specific differences in response to ovarian hormone milieu [291]. In 17β-estradiol-treated rats, increases in the same regions were observed during time-points ranging from 5 to 72 hrs after injections that produced time-dependent fluctuations in 17β-estradiol levels [291]. In 1997, Gibbs showed that 17β-estradiol treatment also produced region specific effects within the basal forebrain which varied with dose and duration of treatment [289]. In fact, Gibbs’ ideas of dose and duration of treatment as mediators of estrogenic effects on the brain and on cognitive function are prevalent now, some 10 years later, are still being pursued and defined, and appear to even be relevant to recent clinical findings such as those from the WHI (discussed earlier in this review). The 1997 study showed, in particular, that MS increases in ChAT-IR were observed after one week of 17β-estradiol treatment at doses of 2, 10, and 25 μg given every other day, but not at a dose of 100 μg, or after 2 or 4 weeks of treatment [289].

Yet, in the NBM, increases in ChAT-IR were only observed after administration of 10 μg of 17β-estradiol for 1 week or 2 μg of 17β-estradiol for 2 weeks [289]. Similar to the attenuated long-term 17β-estradiol effects in rats [289], Gibbs’ and colleagues showed that long-term treatment (2 years) with CEE alone did not alter basal forebrain or hippocampal ChAT or acetylcholinesterase (an enzyme which breaks down acetylcholine) activity in monkeys, but combination therapy (CEE + MPA) decreased both cholinergic markers in the medial septum/diagonal band of Broca of the basal forebrain [292]. However, two years of CEE HT did increase number of cholinergic neurons in the intermediate region (Ch4i) of the nucleus basalis of Meynert in middle-aged monkeys, and this increase was correlated with increasing levels of estradiol [293], an effect not found in young monkeys [293,294]. These findings therefore suggest that estrogens can have differential effects on cholinergic markers in varied basal forebrain regions, and that these effects are transient with lower doses exerting longer-lasting effects on ChAT markers as compared to higher doses. It is possible that the differential effects of 17β-estradiol on the basal forebrain are due to alterations in estrogen receptors (ER), as effects of 17β-estradiol on ER down regulation and recycling are dose- and time-dependent [295,296,297]. Further studies evaluating how timing, referring to ovarian hormone deprivation and estrogen treatment duration, as well as age, impacts efficacy will help determine replacement parameters for optimal brain function during aging. 

In addition to estrogens, other gonadal hormones have also been found to alter basal forebrain cholinergic neurons. Progesterone enhances high affinity choline uptake in several cognitive brain regions, including the frontal cortex [298]. Furthermore, ChAT mRNA levels in the basal forebrain fluctuate with the estrous cycle in the rat, and in Ovx animals progesterone elevated the 17β-estradiol-mediated increase in ChAT mRNA levels relative to Ovx controls [291]. Androgens have also been shown to alter various experimental parameters of basal forebrain cholinergic neurons. Specifically, paralleling Ovx and estrogen treatment findings in female rats [286], Gdx decreases, and subsequent testosterone treatment increases, markers of ChAT in the basal forebrain [299]; this effect was not found in DHT-treated male animals [300]. Together these studies suggest, although somewhat limited as compared to the estrogen literature, that progesterone and testosterone mediate alterations in the basal forebrain cholinergic system. In the case of testosterone, effects may be mediated by testosterone aromatization to estrogen, as DHT was not found to alter basal forebrain markers of cholinergic activity.

### 4.3. Genomic and non-genomic actions

Classically, gonadal hormone effects are exerted via direct receptor-mediated changes in gene transcription; age-related changes in gonadal hormones and such associated hormone receptors have been well studied [e.g., 301]. It is known, for example, that 17β-estradiol mediates its effects by binding to α and/or β estrogen receptors (ERs) in the cytoplasm. Specifically, binding of an estrogen to its intracellular α and/or β receptor causes receptor-ligand dimerization, translocation into the nucleus and complexing with other co-factors in the cell (for review see [302]). The complex can bind directly to an estrogen-response element located on the promoter of specific genes and thereby alter gene transcription. More recently, evidence suggests that gonadal hormones can also be effectors of cellular processes other than those mediated by gene transcription. While little work has been done in this regard for androgens, estrogens have been studied. Indeed, 17β-estradiol is the most widely studied gonadal hormone with respect to receptor-mediated non-genomic actions. Recent evidence indicates that estrogenic effects are influenced by the putative G-protein coupled membrane-bound ER [*i.e*., ER-X, 303] and/or GPR30, a G-protein coupled receptor that responds rapidly to estrogen [304]. It is therefore possible that cognitive effects of 17β-estradiol, and perhaps other gonadal hormones, are mediated partially via membrane bound receptors. In this regard, Gibbs and colleagues [305] recently found that the GPR30 agonist G1 enhanced delayed-match-to-position learning. In addition to effects mediated by ER-X and GPR30, estrogens have also been reported to exert robust non-genomic effects by multiple mechanisms [306]. Further, Frick’s laboratory recently showed that 17β-estradiol enhanced object memory via dorsal hippocampal ERK activation, and identified membrane-bound ERs as mediating 17β-estradiol improvements in memory consolidation [138]. Novel hypotheses have also recently been set forth that there is a coupling of membrane and genomic actions of estrogens and other hormones regarding effects on the central nervous system [307]. Thus, collectively, both genomic and non-genomic actions are likely to contribute to the effects of gonadal hormones on the brain. The work done thus far identifies that non-genomic actions are a contributing mechanism to cognitive effects of gonadal hormones, and that genomic and non-genomic effects could converge. Further research to fully identify the mechanisms of gonadal hormone effects both at the cellular and the systems level in this regard should provide exciting new understandings and perspectives leading the path to healthy cognitive aging. 

### 4.4. Neuroprotective and neurotrophic effects 

A potential mechanism for gonadal hormone-mediated cognitive enhancement is improving neuron viability, perhaps enhancing cognitive functioning via the preservation of related brain circuitry. Work from the Brinton laboratory has shown that 17β-estradiol enhances the growth of certain populations of neurons, possibly via an NMDA-dependent mechanism [308], and CEE enhances the growth and survival of neurons in cognitive brain regions *in vitro* [309,310]. Moreover, CEE protected against β-amyloid-induced neuronal death in rat hippocampal cell cultures, and attenuated ischemic effects of experimental stroke [310,311]. In addition to the compound formulation CEE, certain select components of CEE enhanced markers of neuroprotection [308,312]. The estrogens Δ^8,9^dehydroestrone and equilin were the two primary components of CEE that showed the most consistent and potent neuroprotective effects *in vitro* [312]. More specifically, Δ^8,9^ dehydroestrone exposure attenuated lactate dehydrogenase (*i.e.,* LDH) release and increased adenosine triphosphate (*i.e.,* ATP) production after exposure to the neurotoxins glutamate or β-amyloid^25-35^ [312]. Equilin treatment also demonstrated neuroprotective properties, as it attenuated LDH release after glutamate exposure [312], decreased hippocampal neuronal loss and calcium mobilization after gp^120^ exposure [313] and positively influenced neuronal growth and survival [314]. Lastly, progesterone, alone and in combination with 17β-estradiol, protected hippocampal neurons from glutamate toxicity; however, MPA did not protect against glutamate toxicity, and it attenuated the 17β-estradiol-induced protection when it was co-administered [186]. In fact, another study found that MPA pretreatment exacerbated hippocampal neuronal death via glutamate toxicity [315]. Together, this body of work suggests that 17β-estradiol and CEE can enhance markers of neuronal health and viability, which may be one mechanism for their ability to enhance cognition; this effect is not shared by the commonly prescribed synthetic progestin, MPA. An interesting caveat that requires further scientific inquiry is that while progesterone appears to be neuroprotective *in vitro*, the majority of the evidence suggests that it is not beneficial to cognition. In fact, as discussed above, studies have shown that progesterone impairs cognition when given alone, and that it can reverse the beneficial effects of estrogens on spatial cognition.

Androgens have also been found to be neuroprotective *in vitro*. Testosterone, at physiological concentrations, protected primary human fetal neurons from apoptosis induced by serum deprivation and Abeta1-42 toxicity [316,317]. Additionally, testosterone protected primary rat hippocampal neurons against beta-amyloid toxicity [318], and testosterone protected C1300 mouse undifferentiated neuroblastoma cells against 3-nitro-L-tyrosine (*i.e.,* a reactive nitrogen species) toxicity [319]. An important caveat to this story is that testosterone is readily converted to 17β-estradiol in brain by the aromatase enzyme [320]. Hence, any neuroprotective actions found after androgen treatment may in fact be mediated by 17β-estradiol, as is the case for dehydroepiandrosterone (DHEA) protection after kainic acid toxicity in the rat hippocampus, whereas the aromatase inhibitor fadrozole blocked DHEA’s effect [321]. Many studies do take the aromatization of testosterone into consideration by also treating neurons with androgens that cannot be converted to 17β-estradiol (e.g., mibolerone and DHT); these studies find consistent neuroprotection mediated by androgens that cannot be aromatized, suggesting actions via the androgen receptor [316,317,318]. Thus it appears that androgens, like estrogens, are neuroprotective *in vitro*, an effect that can be mediated by direct androgenic actions via the androgen receptor or through testosterone’s aromatization to 17β-estradiol. 

Neurotrophins may be another mechanism of gonadal hormone-induced neuroprotection and cognitive enhancement. Survival and maintenance of cholinergic neurons are dependent upon neurotrophins, including nerve growth factor (NGF) and brain-derived neurotrophic factor (BDNF), and NGF and BDNF have been associated with cognitive function [35,322,323,324,325,326,327,328]. Ovarian hormones have been found to influence neurotrophin systems. Neurotrophin receptor mRNA levels were shown to fluctuate across the estrus cycle [329]. Also, 17β-estradiol treatment impacts neurotrophins in young and aged Ovx rats, increasing neurotrophin protein mRNA and neurotrophin receptor (e.g., TrkA) mRNA levels in basal forebrain, frontal cortex and hippocampus [286,329,330], and elevating NGF and BDNF protein levels in cognitive brain regions [184]. Interestingly, our laboratory has found that administration of progesterone abolishes not only the beneficial cognitive effects of 17β-estradiol [97], but also the 17β-estradiol-mediated increases in BDNF, NGF and NT3 in the entorhinal cortex (Figure 5) [97,184], a brain region that has major inputs into the hippocampus and that has been shown to have spatial representations in the rat [331]. Taken together, this suggests that 17β-estradiol alters the neurotrophic system (*i.e.,* neurotrophins, and neurotrophin receptor, levels) in cognitive brain regions, which may be one factor mediating 17β-estradiol’s ability to enhance cognition, and that progesterone also impacts this system. 

Similar to estrogen, testosterone has been shown to affect neurotrophin protein and receptor levels in young rodents [332,333]. We found increases in NGF protein, and decreases in BDNF protein, in the hippocampus and entorhinal cortex of male rats 4-7 months of age, concomitant with elevations in testosterone levels [322]. In various models, testosterone treatment enhances NGF and BDNF expression [334,335,336]. Testosterone treatment also increases NGF expression in the cortex and hippocampus of male rats after hypoxic-ischemic brain damage [336], and the anabolic steroid nandrolone, as well as testosterone, treatments increase NGF levels in hippocampus and septum [333]. While these studies suggest that androgens likely alter neurotrophin levels, whether these relations between androgens, neurotrophins, and their receptors are related to the cognitive effects of androgens remains to be determined. Indeed, there has been little work done evaluating androgens, neurotrophins and cognition in the same study. 

It is known that age-associated memory impairment is coupled with alterations in the cholinergic system [e.g., 347,348,349], and neurotrophin systems have been found to change with age. Specifically, it is thought that with aging and neurodegenerative disease there is deficient retrograde transport of NGF leading to an up regulation of NGF in specific brain regions to compensate [340]. We have shown that neurotrophins correlate with cognitive performance in aged rats, with higher levels of NGF and BDNF protein within the hippocampus and cortex related to worse memory performance in ovary-intact aged female rats [35,341]. Additionally, it is hypothesized that neurotrophin levels increase after brain insult in order to repair and support damaged tissue. This tenet is supported by empirical research finding that neurotrophins promote brain graft survival, cholinergic survival after insult, and cholinergic system enhancement [e.g., 324,342,343]. Neurotrophin supplementation has also been found to ameliorate memory defects in the older rat [19,344,345,346,347,348]. Hence, age-related changes in cognition may be partially mediated by age-related alterations in neurotrophins. That gonadal hormones also impact the neurotrophic system provides exciting avenues of future research which will likely yield insight into the interactive effects of gonadal hormones, memory and aging.

## 5. Scopes of Research within the Tertiary Model: Prior Journeys, Future Directions

Collectively, the research reviewed here provides exciting new avenues for neuroscientists to act as cartographers, mapping new insights and prospects to enhance cognitive health during aging. The research demonstrates that parameters dictating hormone treatment efficacy are complex. In addition, a complete risk-to-benefit ratio should be considered from a multiple systems approach since cardiovascular disease, stroke, sexual dysfunction, osteoporosis, cancer, and diabetes are also influenced by gonadal hormone milieu [see 168]. Even while noting the importance of a “big picture perspective” of the risk-to-benefit effects of hormone therapies, it is clear that gonadal hormones are now known to be important for more than the classical reproductive actions first methodically evaluated by Beach in 1947 [64]. As detailed in this review, estrogens, progesterone and androgens, or “internal secretions”, as discussed at the beginning of the 20th century, are mediators of neural plasticity and brain functions. Over the last decade, both the human and animal literature evaluating the potential influence of gonadal hormones on brain health and function during aging have increased in breadth and depth. Much of this interest stems from clinical findings suggesting hormones impact brain health during aging and in neurodegenerative disease. In the last several years, the importance of understanding the effects of gonadal hormone loss and treatment, including the clinical implications of these insights, has received tremendous interest as never before. Some of this recent enthusiasm is due to realization that the life expectancy of women has increased, yet the age of spontaneous menopause has remained stable, so women are now living approximately one-third of their lives in a menopausal state. This interest is compounded by the fact that it is not clear what ovarian hormone loss, nor ovarian hormone therapy/replacement, does to the brain and its function. However, lack of complete clarity of these complex interactions does not mean we, as a field, have little knowledge of the effects of gonadal hormones during aging. On the contrary, as evidenced by the research discussed in this review, the emerging findings in the literature have been illuminating, diverse, and exciting. Like an explorer looking for new treasures by using information from others before them, a compass and the north star for direction, scientists today are building on older research treasures to guide them to the ultimate destination, a clear understanding of how hormones, cognition and age interact so that we can optimize treatment plans to promote healthy brain aging. 

So far, research has shown that multiple parameters impact the extent, and even the direction, of cognitive effects of gonadal hormones. Overall, animal research has given much insight into clinical findings in this regard. For example, animal research suggests that estrogen therapies can benefit cognition, but these favorable effects depend on key factors including timing of hormone therapy initiation, age, dose, and specific estrogen type. Indeed, rodent findings indicate that estrogen therapy initiation should coincide with onset of ovarian hormone loss, as longer periods of hormone deprivation and older age each attenuate the cognitive efficacy of treatment. Further, mnemonic benefits of estrogen-including hormone therapies are influenced by progestin co-administration. Progestins counteract some beneficial cognitive and trophic effects of estrogen treatment, and unopposed progestins have been implicated in cognitive deficits. However, women with an intact uterus taking estrogens must also take a progestin to decrease the risk of endometrial hyperplasia [187,349]. Optimization of hormone therapy includes discovering progestins that do not adversely impact cognition, but mitigate the negative impact on the uterus. Estrogenic treatments specifically targeting the brain, thereby obviating the need for co-administration of a progestin, are also promising. Androgens have also been shown to alter cognition. In general, androgen treatment provides cognitive benefit in males and females, although research in this realm is limited. Thus far, the work suggests that efficacy depends on the specific type of androgen administered, as well as dosage since effects may subserve a curvilinear function.

While much has been discovered regarding relationships between aging, spatial learning and memory, and gonadal hormones, the compass for future aging research points toward clarifying the interactions that exist between these three key points. The need for interdisciplinary evaluations to define details of these complex interactions is key. To broaden our understanding of how these tenets interact, further evaluations of the neurobiological mechanisms within brain regions involved in memory, and influenced by aging and gonadal hormones, should be on the forefront of scientific explorations. Animal models are a promising path as they allow methodical experimental evaluation while controlling for, or obviating, factors likely influencing outcome in clinical studies such as exact age, duration of hormone loss or replacement, specific type and dose of hormone manipulated, socioeconomic status, and education. Rather, in animal models many of these issues, and interactions thereof, can be systematically tested and used to compliment and aid understanding of the clinical studies. Indeed, the research presented here provides abundant evidence that animal models are useful in identifying effective nootropic therapies, as well as limitations of therapies. Within this context as well, understanding the limitations of animal models, and discovering new options to improve interpretability and translatability to humans, will help build the path to optimal cognitive aging for men and women. 

## 6. A Final Comment: How Understanding the Crossroads of Gonadal Hormones, Memory, and Age Can Help Draw a Map to Optimal Brain Aging

Here, we discussed a tertiary model representing interactions between aging, spatial learning and memory, and gonadal hormones given that: 1) gonadal hormones change with age, 2) aging impacts spatial learning and memory, and 3) gonadal hormones affect spatial learning and memory. The gonadal hormones estrogens, progesterone, and androgens each impact spatial learning and memory, and very recent evidence suggests that aging alters responsivity or sensitivity to these hormones in the context of spatial cognition and postulated underlying neurobiological mechanisms. Hippocampal-dependent spatial memory is particularly susceptible to age-related decrements, especially when task demand or load is elevated. Gonadal hormones can substantially alter spatial ability, and are potent modulators of brain structure and function in brain regions known to modulate spatial learning and memory. Many of these cognitive brain regions are affected by aging.

Lifetime experiences, exposures, and differences in hormonal milieu across individuals contribute to an aged population that is heterogeneous with diverse cognitive outcomes. The latitude of one’s brain plasticity can be interpreted within a framework of allowing one the ability to “optimize their aging” and find their own map to cognitive health as aging ensues. Identifying the various components of the complex interactions between gonadal hormones, memory, and age, including detailed evaluations using basic science, systems, and clinical evaluations, is the optimal approach to converge the many findings that appear contradictory. In fact, as new data emerge it will likely become clear that findings are not contradictory at all. Rather, they may be dependent on numerous variables not yet taken into account in many studies, because these variables that are so very important to outcome are not yet discovered. By aligning basic scientific discoveries with clinical interpretations, we can capitalize on opportunities for these discoveries and subsequent interventions so that individuals can maximize their potential for brain health as aging ensues.
